# Cobalt Oxide Nanoparticles Stabilized by d‑Glucose: Synthesis, Magnetic Properties and Catalytic Activity for Hydrogen Generation from NaBH_4_ Hydrolysis

**DOI:** 10.1021/acsomega.6c00197

**Published:** 2026-05-19

**Authors:** Leonardo C. Moraes, Anderson S. Paschoa, Leonardo A. da Silva, Rodrigo Ribeiro-Andrade, Renzo Rueda-Vellasmin, Edson C. Passamani, André E. Nogueira, Humberto V. Fajardo, Cleber C. Figueredo

**Affiliations:** † Laboratório de Química Inorgânica, 681196 Universidade do Estado do Amapá, Avenida Presidente Vargas, 650, Centro, Macapá, Amapá 68900-070, Brazil; ‡ Departamento de Física, 28126 Universidade Federal do Espírito Santo, Avenida Fernando Ferrari, 514, Goiabeiras, Vitória, Espírito Santo 29075-910, Brazil; § Departamento de Química, Instituto de Ciências Exatas e Biológicas, 28115 Universidade Federal de Ouro Preto, Rua Diogo de Vasconcelos, 122, Pilar, Ouro Preto, Minas Gerais 35400-000, Brazil; ∥ 28114 Centro de Microscopia da Universidade Federal de Minas Gerais, Avenida Antônio Carlos, 6627, Pampulha, Belo Horizonte, Minas Gerais 31970-901, Brazil; ⊥ Departamento de Química, Divisão de Ciências Fundamentais, 74360 Instituto Tecnológico de Aeronáutica, Praça Marechal Eduardo Gomes, São José dos Campos, São Paulo 12228-615, Brazil; # Departamento de Botânica, Instituto de Ciências Biológicas, Universidade Federal de Minas Gerais, Avenida Antônio Carlos, 6627, Pampulha, Belo Horizonte, Minas Gerais 31970-901, Brazil

## Abstract

Hybrid CoO_2_@CoO nanoparticles were synthesized for the first time using d-glucose as a stabilizing agent. Their structural, morphological, vibrational, and magnetic properties were systematically studied using a wide range of techniques, such as Transmission Electron Microscopy (TEM, HR-TEM, EDS), X-ray Photoelectron Spectroscopy (XPS) and, DC and AC magnetic magnetization experiments (M­(T), M­(H), and χ­(*T*,*f*)). XPS data indicated high chemical stability, while magnetic measurements revealed that the hybrid material exhibited magnetic ordering below 8 K. The susceptibility peak showed a dependence on the excitation-field frequency, a feature commonly found in ensembles of small magnetic particles in the superparamagnetic regime. These nanoparticles were successfully used as catalysts for hydrogen generation via NaBH_4_ hydrolysis. Their high catalytic activity sites were attributed to their well-distributed crystallites, which are responsible for the highly dispersed catalytic that promotes the hydrolysis of alkaline sodium borohydride solution. The hydrogen generation rate reached 1452.5 mL H_2_ min^–1^ g_cat_^–1^ at room temperature (25–30 °C) and the apparent activation energy was calculated to be 51.73 kJ mol^–1^.

## Graphical abstract

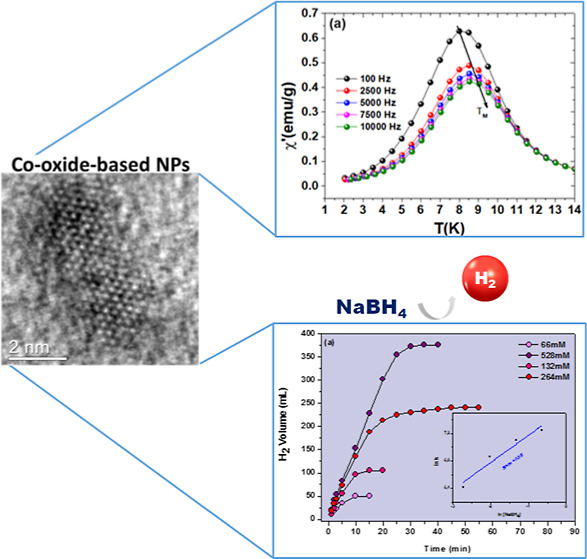

## 1 Introduction

Magnetic nanoparticles (MNPs) have attracted considerable interest due to their applications in a wide range of fields, including data storage, environmental remediation, biomedical imaging, diagnostics, anticancer therapy, and heterogeneous catalysis.^1,2^ In catalytic systems, their magnetic properties offer a key advantage: they can be easily recovered using an external magnetic field and reused, which contributes to the sustainability of the process.^3^ Among MNPs, cobalt-based nanoparticles (Co-NPs) are currently used for all the applications mentioned above. It is therefore essential to consider the synthesis parameters (reaction conditions, temperature, solvent, etc.), as they significantly influence the structural, chemical, and magnetic properties of the resulting nanoparticles.^4^

The synthesis of MNPs typically involves the formation of a metal core stabilized by molecules (called ligands) that also determine the shape, surface valence state, reactivity and physical properties of the synthesized ensemble of nanoparticles (NPs). A wide variety of molecules can be used as ligands during the synthesis of NPs, resulting in a high diversity of MNPs and, consequently expanding their potential applications.^5^ In this context, materials science researchers are continuously exploring new ligands for the synthesis of MNPs and investigating the properties and applications of the resulting materials.

Among the wide range of MNP applications, an important aspect to consider—given the growing emphasis on sustainability—is the development of synthesis methods that are scalable (with good sample reproducibility), low-cost, and environmentally friendly. In this context, we explored the use of glucose, a naturally abundant biomolecule, as a stabilizer ligand,^6^ employing a simple and economically attractive synthesis of Co-NPs with potential applications in several fields.

Specifically in the field of catalysis, the agents involved in hydrogen production are highly valued, as this product is considered one of the main alternatives to fossil fuels, the burning of which has resulted in intense CO_2_ pollution and climate change.^7–10^ Water and biomass are among the main renewable resources used for hydrogen production. For the former, water splitting is promoted by electrolysis,^11,12^ whereas gasification is used due to higher efficiency and positive effects for the environment.^10^ Although promising, these methodologies still face economic and technical challenges.^13^

In parallel to these approaches, chemical hydrogen storage materials, particularly hydrides, have emerged as attractive alternatives due to their ability to release hydrogen in a controlled and on-demand manner. Hydrides have been considered excellent hydrogen carriers.^14^ Among all of them, sodium borohydride (NaBH_4_–hydrogen storage capacity of 10.8 wt %) seems to be the best to release a high yield of pure hydrogen upon its hydrolysis described by eq 1.^15–17^

1 $${NaBH}_{4}+{2H}_{2}O\to {NaBO}_{2}+{4H}_{2}$$

The NaBH_4_ aqueous solution seems to be an ideal hydrogen source due to its stability, nonflammability, and nontoxicity.^18^ The NaBH_4_ hydrolysis process also offers some advantages, such as operating at mild temperatures and generating an environmentally safe byproduct (NaBO_2_) that can be recycled to generate the reactant. Although there are still some challenges, the future for this technology is promising.^19^ At ambient conditions, the NaBH_4_ self-hydrolysis is spontaneous but rather slow. However, this process can be significantly accelerated by the presence of a catalyst.^20^

The hydrolysis reaction can be promoted by homogeneous or heterogeneous catalysts, with the latter generally being preferred because it can be more easily separated from the reaction medium and reused.^19–21^ Among the catalysts for hydrolysis of sodium borohydride, those containing noble metals show excellent activity. However, their high cost represents a significant limitation. As a more economical alternative, non-noble metal-based catalysts emerge as strong contenders, and cobalt-containing catalysts seem to be one of the best options.^19–21^ Some cobalt catalysts exhibit catalytic activities comparable to those presented by noble metals. Thus, herein we report, for the first time, the synthesis of cobalt-oxide-based nanoparticles (Co_1–*x*_O_*x*_-based NPs) stabilized by d-glucose and a full characterization of their structural, morphological, vibrational and magnetic properties, as well as their application as a heterogeneous catalyst in the hydrolysis of NaBH_4_ for hydrogen generation. The synthesis strategy used in this work was based on the use of anhydrous d-glucose as a green and accessible stabilizing ligand for Co_1–*x*_O_*x*_-based NPs. The reduction of cobalt-(II) chloride hexahydrate in aqueous medium under ambient atmosphere, in the presence of a stoichiometric amount of glucose, led to the formation of well-defined Co-oxide nanostructures. This ligand-mediated approach proved effective in controlling particle shape, size distribution, and surface characteristics. As discussed in the literature,^22,23^ the nature and amount of the stabilizing agent play a decisive role in the nucleation and growth of metal-based nanostructures. In our system, CoO_2_@CoO-NPs were obtained following simple aqueous processing and purification via successive washing with distilled water. The result highlights the potential of glucose not only as a reducing/stabilizing agent but also as a key factor in enabling the formation of a metastable cobalt oxide phase under mild and environmentally benign conditions. Therefore, the magnetic properties of the synthesized hybrid CoO_2_@CoO-NPs represent a crucial advantage for their application in NaBH_4_ hydrolysis. Their magnetic responsiveness enables efficient recovery from the reaction medium through an external magnetic field, minimizing catalyst loss and facilitating reuse. Combined with the high chemical stability and high active sites provided by the nanostructured morphology, these features enhance catalytic performance while contributing to a more sustainable and economically viable hydrogen generation process.

## 2 Materials and Methods

### 2.1 Synthetic Procedures

In a Falcon tube, cobalt­(II) chloride hexahydrate (Sigma-Aldrich, 98%), CoCl_2_·6H_2_O (50.0 mg, 0.21 mmol), anhydrous d-glucose (Sigma-Aldrich, 96%), C_6_H_12_O_6_ (37.8 mg, 0.21 mmol) and distilled water (12 mL) were added. Sodium borohydride (Sigma-Aldrich, 98%), NaBH_4_ (4.0 mg, 0.11 mmol) was then added in a single portion under stirring. The reaction mixture was left to stand at room temperature (RT) for 60 min. After this period, the resulting gray suspension was concentrated by centrifugation at 3600 rpm for 10 min. The obtained dark solid was washed with distilled water (3 × 5 mL) and dried under vacuum, affording the corresponding cobalt oxide nanoparticles. This procedure was repeated three times under identical experimental conditions to evaluate the reproducibility of the synthesis. The obtained materials exhibited consistent properties, with variations within acceptable limits, confirming the efficiency of the synthetic route.

### 2.2 Characterization Techniques

The presence of Co-oxide core NPs was confirmed by conventional characterization techniques. Transmission Electron Microscopy (TEM) analyses were performed on a FEI Tecnai G2-20 S-TWIN operating at 200 kV with a point resolution of 1.9 Å. The determination of the particles’ mean size was made through a manual analysis of enlarged micrographs by measuring ca. 200 particles on a given grid. Just before TEM observations, a drop of the NPs dispersion was deposited on a copper grid with a reticulated amorphous carbon film and allowed to dry. The structure and elemental composition of the hybrid CoO_2_@CoO-NPs, at the nanoscale, were analyzed by High-Resolution TEM (HRTEM), while the EDS (Energy Dispersive Spectroscopy) point acquisitions were performed by using a Silicon Drifted Detector (SDD) from Oxford Instruments. For X-ray Photoelectron Spectroscopy (XPS) analysis, the Thermo Scientific K-alpha instrument was employed, and the binding energy of the spectra was referenced to the C-1s peak at 284.8 eV, with data analysis performed using Casa XPS version 2.3 software.

### 2.3 Measurement of Magnetic Properties

Magnetic properties of CoO_2_@CoO-NPs were recorded using a Physical Properties Measurement System Evercool-II setup from Quantum Design Inc., operating respectively with a vibrating sample magnetometer (VSM option) mode, and by implementing the AC susceptibility method, and where the AC measurements were performed in a range of frequencies of 2.5–10 kHz with an oscillating probe field of 10 Oe in a temperature region of 2–20 K. M­(T) curves were obtained in zero-field cooling (ZFC) and field cooling (FC) protocols with a probe field of 10 Oe, while ZFC M­(H) loop at 4 K was obtained for scan field of ± 50 kOe (or μ_o_ *H* = ± 5 T).

### 2.4 Catalytic Hydrolysis of NaBH_4_

The hydrogen, generated from sodium borohydride hydrolysis, was quantified using the classical water volume displacement technique. In a typical experiment, the reaction solution containing sodium borohydride (NaBH_4_) and sodium hydroxide (NaOH) was introduced into the reactor, which was kept inside a water bath to maintain the reaction temperature controlled. Then, an appropriate amount of the CoO_2_@CoO-NP catalyst was loaded into the reactor to initiate the reaction under continuous stirring. The cumulative hydrogen volume was periodically recorded and represented graphically as a function of time. The effects of reaction parameters, such as catalyst mass (5–15 mg), NaOH concentration (2.5–20 wt %), NaBH_4_ amount (0.025–0.2 g) and temperature (10–55 °C) on the hydrogen generation rate (HGR, expressed by mLH_2_ min^–1^ g_cat_^–1^) were evaluated. The HGR was determined by the linear fit of the obtained hydrogen evolution curves. The activation energy for the hydrolysis reaction catalyzed by CoO_2_@CoO-NPs was determined using the logarithmic form of the Arrhenius eq (eq 2).

2 $$\ln\left(k\right)=\ln(A)-\frac{E_{a}}{RT}$$

In eq 2, *k* is the reaction rate constant, *A* is frequency factor, *T* is the absolute temperature (in Kelvin), *R* is the universal gas constant (8.314 J mol^–1^ K^–1^) and *E*_a_ is the activation energy (kJ mol^–1^).

The reusability of the synthesized catalyst in NaBH_4_ hydrolysis was assessed over three consecutive cycles under identical reaction conditions. After each cycle, the catalyst was separated from the reaction mixture. The magnetic capacity of the catalyst makes the separation process more efficient, since considerable aggregation of the NPs occurs on the stirring bar due to magnetic attraction, facilitating the reuse of the material. The catalyst was washed multiple times with distilled water, dried at 70 °C during 12 h, reweighed, and reused in the next cycle.

## 3 Results and Discussion

### 3.1 Characterization of Cobalt Oxide Nanoparticles

The TEM image of Co-oxide-based NPs (Figure 1a) shows discrete black points homogeneously distributed in a matrix, indicating that the as-prepared NPs were well-dispersed and exhibit crystalline features with minimal agglomeration. Figure 1b shows that the NPs exhibit a narrow particle size distribution (PSD), with an average size of 8 (±4) nm, and the largest particles (ca. 20 nm) being rare. The small size and good distribution NPs are characteristics that favor weak magnetic dipolar interactions and enhance surface contributions to catalytic activities,^24^ suggesting a good potential for these Co-oxide-based NPs.

**Figure 1:**
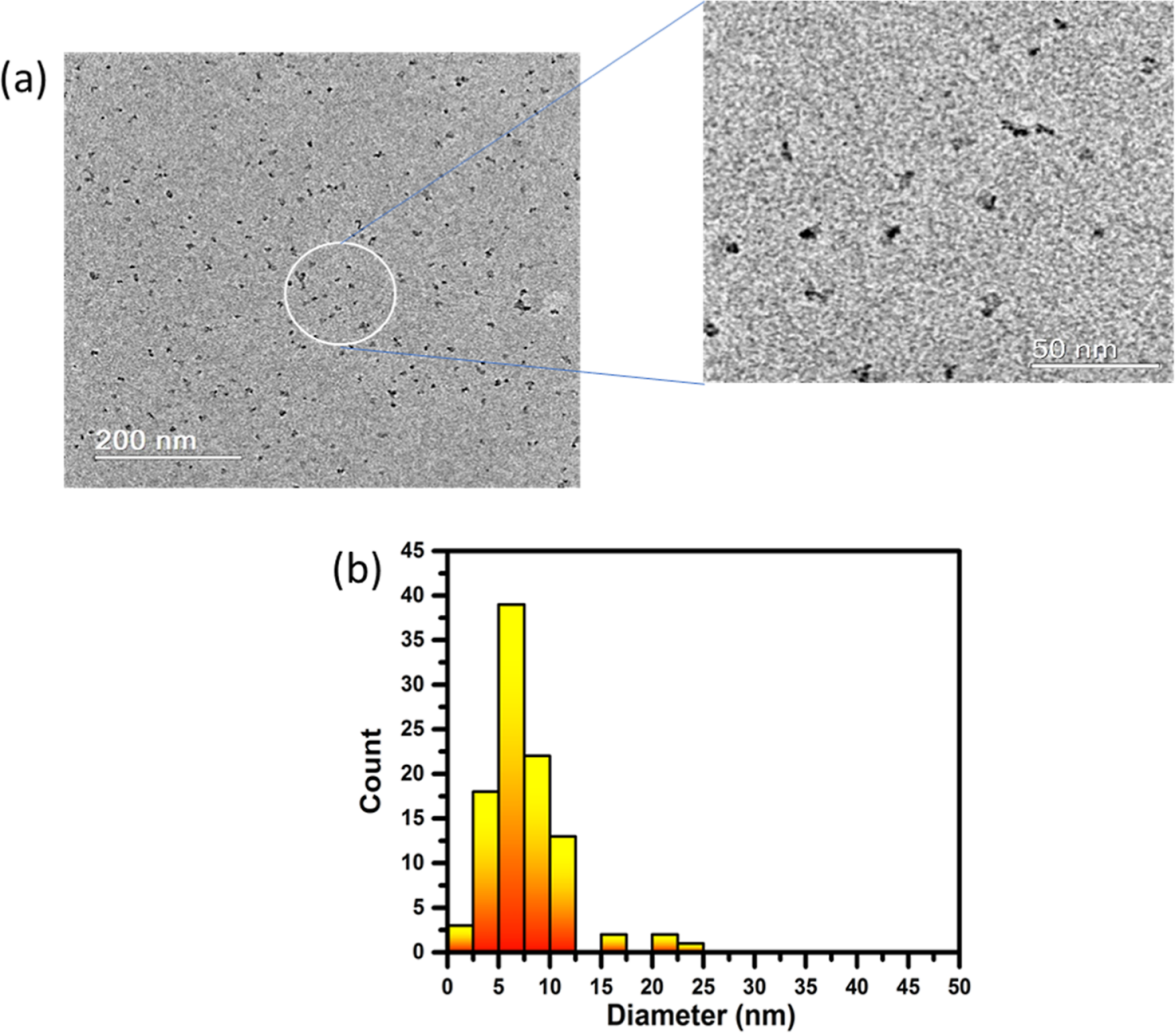
TEM images with zoomed inset (a) and particle size distribution histogram of Co-oxide-based NPs (b).

The crystal structure and chemical composition of the Co-based NPs were determined by High-Resolution TEM and scanning modes (coupled with EDS). Figure 2a,c display the HR-TEM images of the NPs, while their Fast Fourier Transforms (FFT), shown in Figure 2b,d, were calculated to determine the interplanar spacings and crystalline structure. The interplanar spacings corresponding to the (010) and (111) planes were found to be 2.4 Å and 2.1 Å, respectively, while the angles between these planes were measured as 52° and 71°, respectively. A detailed investigation of the FFT revealed that these NPs are predominantly associated with the CoO_2_ phase oriented along the [001] zone axis, which also corresponds to the orientation exhibited in Figure 2b,d.

**Figure 2:**
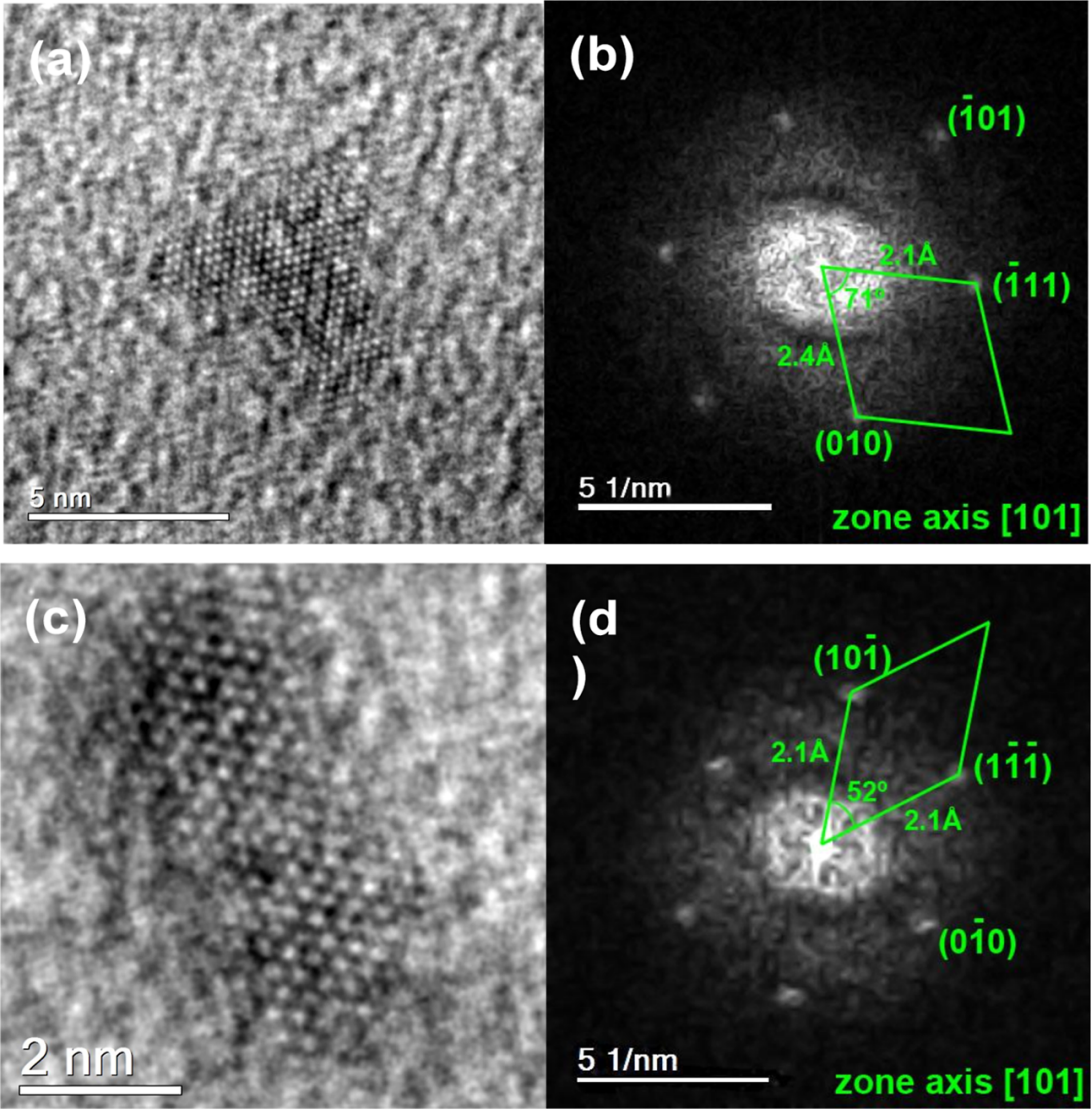
(a,c) HRTEM and (b,d) FFT image of the Co-oxide-based NPs oriented along [001] zone axis.

To confirm the NPs structure, we performed a selected area electron diffraction (SAED), in which the diffraction from a collection of Co-oxide-based NPs is evaluated. Figure 3a shows the SAED image with the typical polycrystalline distribution of NPs, consistent with the random distribution of orientations of NPs in the TEM grid. To analyze the SAED image, the intensity profile of the rings as a function of their distance *q* (1/nm) from the center of the diffraction pattern (DP) was calculated, as shown in Figure 3b by the red curve. The background subtracted intensity profile is shown in Figure 3c (blue curve). To confirm the structure and composition of the Co-oxide-based NPs, crystallographic information corresponding to different Co and O phases was obtained from the Crystallography Open Database (COD) and used to calculate the interplanar distances and rings intensity in the DP. After a thorough phase analysis, comparing the simulations with the experimental results, it was determined that the CoO_2_ structure cataloged in file no. 1522027 best fits the experimental data,^25^ as displayed in Figure 3c.

**Figure 3:**
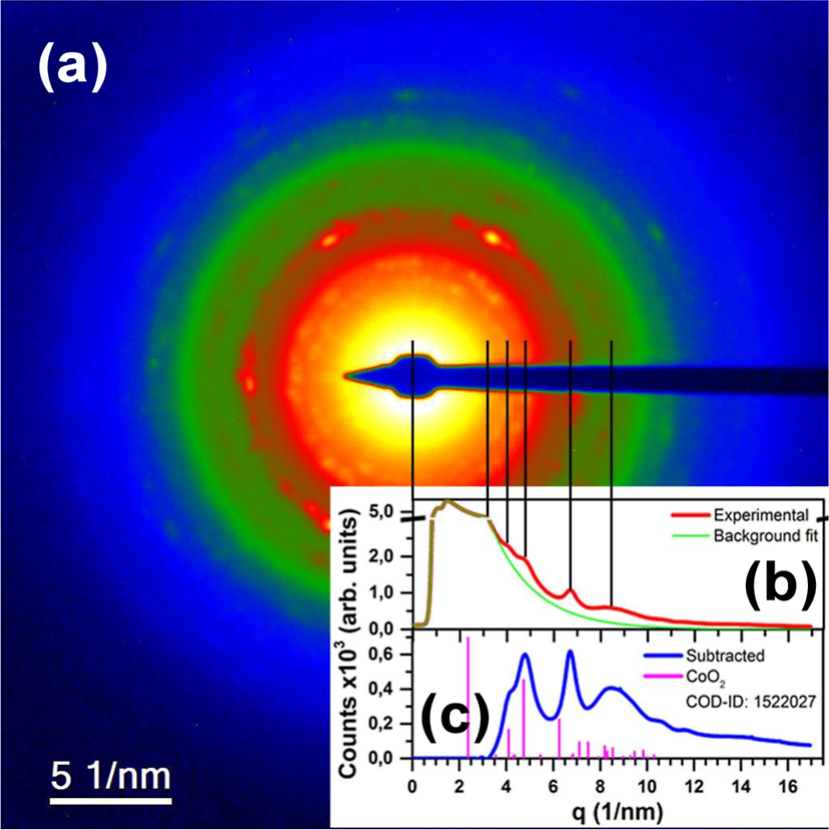
(a) Co-oxide-based NPs SAED image used to obtain the profile intensity of the rings shown in (b) and the background subtracted intensity profile in (c).

Additional chemical analyses by EDS were carried out to validate the previous conclusions and to identify their chemical composition. Figure 4a shows the EDS analysis of the NPs indicated in HR-TEM image (Figure 4b). The EDS results indicate the presence of the Co, O, C, and Cu, with the last two elements assigned to the TEM grid. Although a minor portion of the O signal results from the TEM grid, Co and O were determined to be 28 and 72%, respectively, after the TEM grid signal had been removed from the NP signal, resulting in a value similar to those related to CoO_2_ composition. This result agrees with those found previously from the SAED data. Thus, we can assume that our nanoparticles have a CoO_2_ core, but their surface should be investigated.

**Figure 4:**
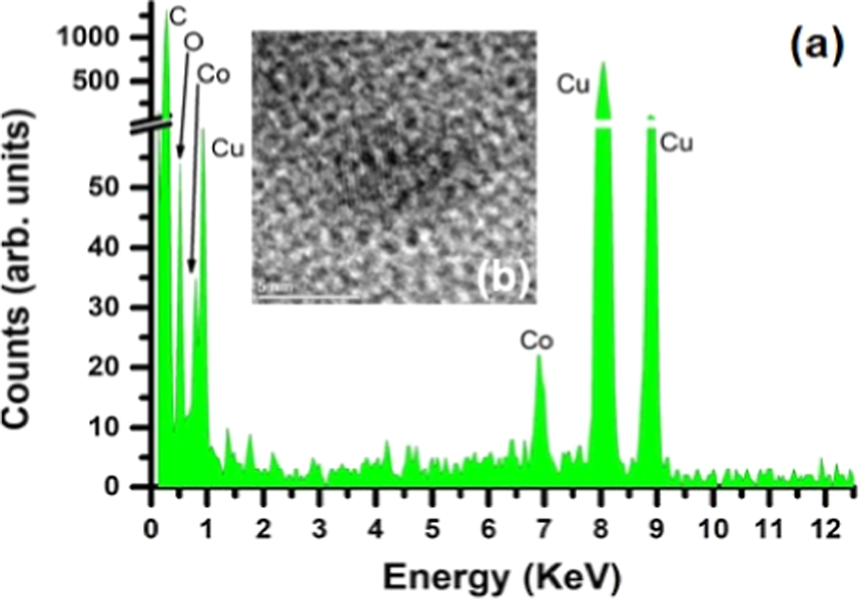
(a) EDS spectrum of NPs and (b) HR-TEM image of a specific nanoparticle.

Additional information on the surface elemental composition and the oxidation states of cobalt ions in the NPs was provided by XPS analysis (Figure 5). The survey spectrum revealed characteristic signals of cobalt (Co), oxygen (O), and carbon (C), the latter attributed to organic residues originating from the use of the d-glucose as a stabilizing agent during synthesis. In addition, the presence of boron (B) was also detected, originating from the sodium borohydride used in the reduction of the cobalt precursor salt.

**Figure 5:**
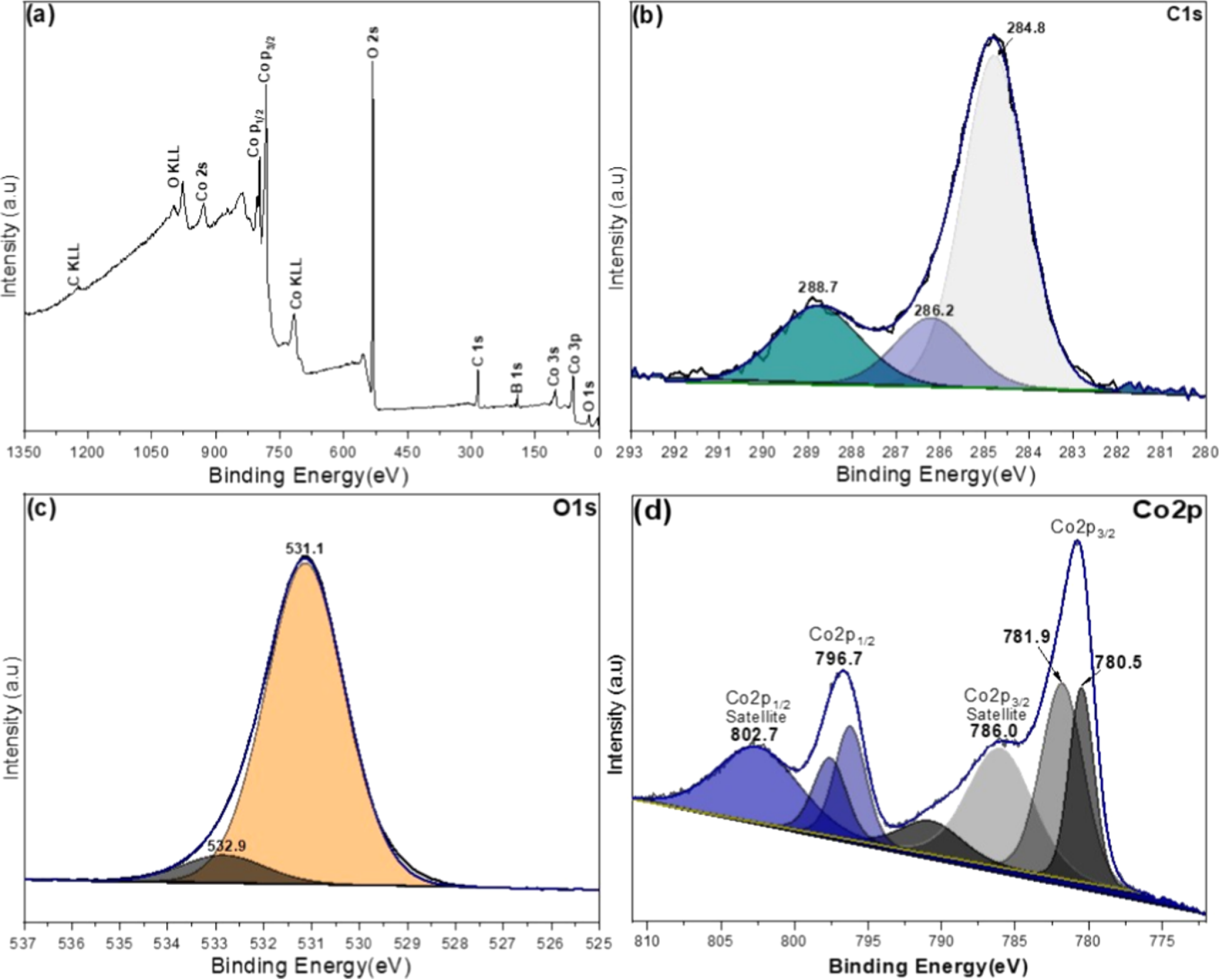
XPS survey spectrum (a) and high-resolution XPS spectra of C 1s (b), O 1s (c), and Co 2p (d) of the hybrid CoO_2_@CoO-NPs.

High-resolution C 1s analysis (Figure 5b) revealed components centered at 284.8 eV (C–C/C–H), 286.2 eV (C–O), and 288.7 eV (O–C=O), consistent with surface organic contaminants or residual glucose from the stabilizer. In the O 1s region (Figure 5c), a main peak was observed at 531.1 eV, with a shoulder at 532.9 eV, which can be attributed to hydroxyl groups, carbonate oxygen, or adsorbed water molecules. These values are shifted relative to the binding energy of lattice oxygen typically found in highly oxidized cobalt oxides, such as CoO_2_, which generally appear at lower binding energies (∼529.5–530.0 eV).^26^

The high-resolution Co 2p spectrum (Figure 5d) exhibits main peaks at 780.5 and 781.9 eV (Co 2p_3/2_), along with their corresponding satellites at 786.0 eV, and Co 2p_1/2_ peaks at 796.7 and 802.7 eV. This spectral profile is characteristic of the Co^2+^ state, as observed in the Co_3_O_4_ and CoO phase, which is distinguished by the presence of intense satellite features and the absence of significant signals around 779 eV, as typically found in Co^3+^ species (absence of Co^3+^ valence state rules out the presence of the Co_3_O_4_ and Co_2_O_3_ phases). The shape and position of the satellites further support the assignment of Co^2+^ as the predominant oxidation state, suggesting that the surface of the Co-oxide NPs is primarily composed of CoO.^26^

In the deconvolution of the Co 2p region, an additional low-intensity feature was identified at approximately 790.8 eV. Although this peak is not directly associated with the main Co 2p_3/2_ or Co 2p_1/2_ components, its position is consistent with secondary shakeup transitions commonly observed in Co^2+^-containing compounds under heterogeneous chemical environments.^27^ This interpretation is further supported by the presence of various surface species, including hydroxyls, carbonates, and organic residues.

While the XPS data predominantly indicate the presence of the Co^2+^ state on the NP surfaces, characteristic of CoO, a CoO_2_ phase was detected by structural analyses performed by HR-TEM. Additionally, energy-dispersive X-ray spectroscopy revealed a Co/O atomic ratio close to 1:2, in agreement with the stoichiometry of CoO_2_. The absence of clear CoO_2_ signals in the XPS spectra is attributed to the surface sensitivity of the technique, which probes only the outermost layers of the material. Given that CoO_2_ is a metastable phase, it is susceptible to surface reduction upon exposure to ambient conditions or even during X-ray irradiation. As a result, it is plausible to assume a core–shell-like model, where the nanoparticle cores retain the CoO_2_ structure, whereas their surfaces are predominantly composed of CoO. This core–shell model may yield complex magnetic properties because CoO is typically antiferromagnetic (AF) below its Néel temperature of 291 K, while the CoO_2_ phase may show different magnetic behavior (ferromagnetic (FM) or AF or even nonmagnetic/superparamagnetic (SPM)), depending on particle size, shape and crystal structure, factors related to sample preparation.

### 3.2 Magnetic Properties

The magnetic properties of hybrid CoO_2_@CoO-NPs are shown in Figure 6. Below 8 K, the ZFC and FC M­(T) curves (Figure 6a) show magnetic irreversibility. In other words, while a well-defined peak at approximately 8 K is found in the ZFC protocol, a plateau-like behavior is observed for the FC M­(T) curve. Above 8 K, the ZFC and FC M­(T) curves display a similar trend and present the expected Curie–Weiss-like behavior. In addition, for temperatures above 15 K, the magnetization reaches negative values, indicating the presence of a diamagnetic contribution from organic phases (this contribution appears to be important only at low fields and high temperatures).

**Figure 6:**
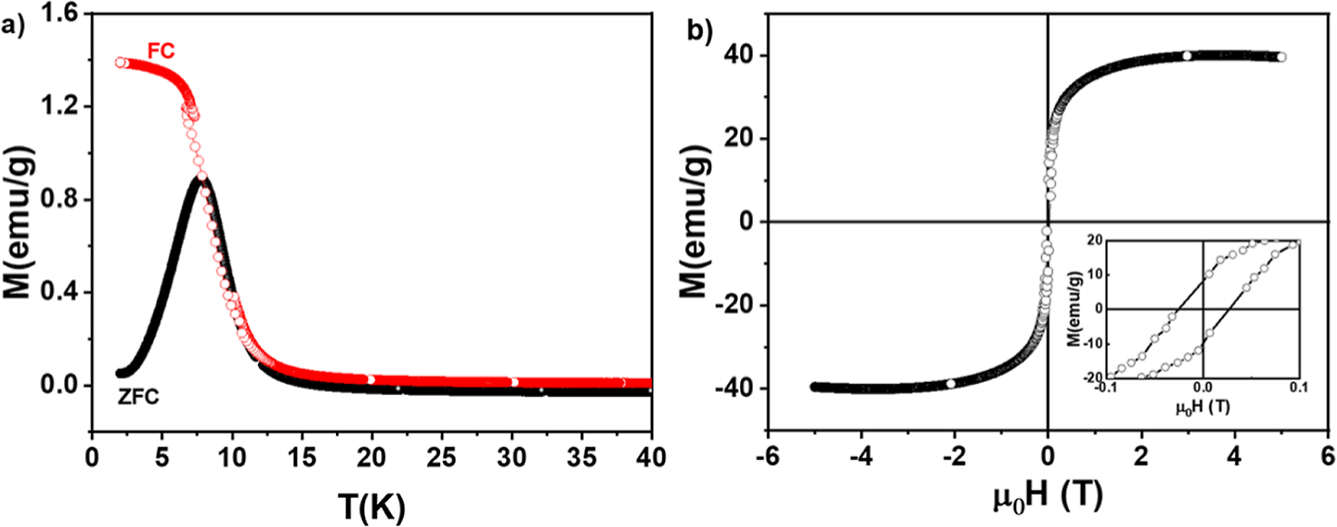
(a) ZFC and FC M­(T) curves for the hybrid CoO_2_@CoO-NPs measured in an applied magnetic field of 10 Oe. (b) 4 K M­(H) loop of this sample. Inset in (b) shows the presence of H_C_ field found in FM materials.

The inverse susceptibility (χ^–1^ = H/M) displayed in Figure 7 also follows the Curie–Weiss law given by 1χ=T−θWC , where the Curie constant is given by $C=\frac{{\mu_{ef}^{2}\mu }_{0}}{3k_{B}}$ with *k*_B_ being Boltzmann constant, μ_o_ the vacuum magnetic permeability, μ_ef_ the effective magnetic moment, and θ_W_ the Curie–Weiss temperature, which defines magnetic interactions among magnetic ions (θ_W_ > 0 for FM interactions, θ_W_ < 0 for AF interactions, and θ_W_ = 0 for no exchange interactions). Fitting the experimental data of Figure 7 with a linear function, we found a value for θ_W_ ≈ 12 K, suggesting FM interactions in CoO_2_@CoO-NPs. An effective magnetic moment μ_ef_ of 9.11 × 10^3^ μ_B_ was obtained, which is similar to the value determined by fitting the 4 K M­(H) loop (Figure 6b) with Brillouin function. This high μ_ef_ value indicates that the magnetism originates from the nanoparticles.

**Figure 7:**
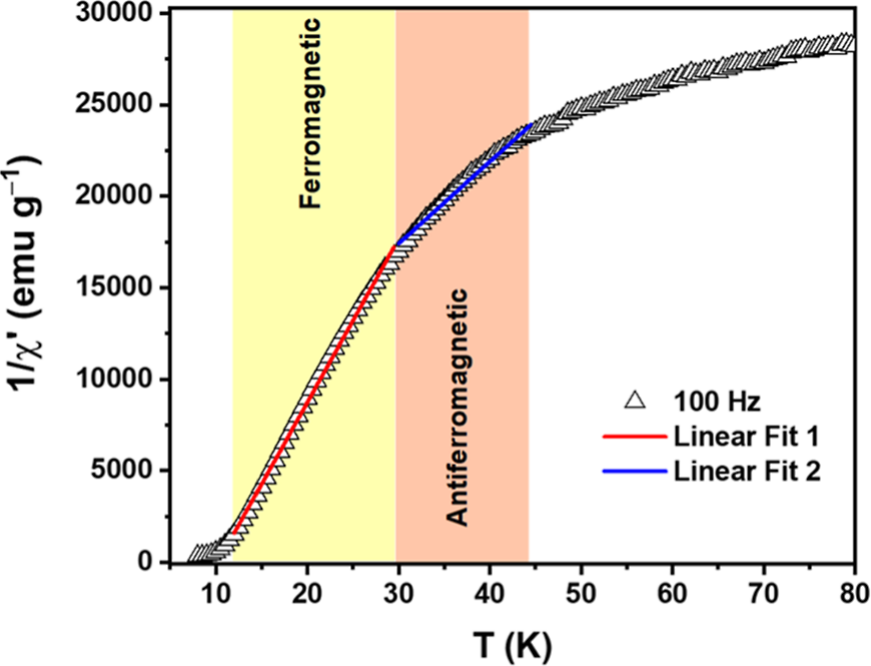
Inverse of susceptibility as a function of temperature for the hybrid CoO_2_@CoO-NPs. The solid lines are results of fittings using the Curie–Weiss law. The red solid line corresponds to the ferromagnetic contribution of CoO_2_, while the blue line may indicate some contribution from the antiferromagnetism of CoO.

On the other hand, considering that CoO is often found in AF state, we measure predominantly the magnetic response from the CoO_2_ cores. Figure 6b also confirms the FM character of the hybrid CoO_2_@CoO-NPs, i.e., it presents a hysteresis (nonzero coercivity field (H_C_)) and magnetization saturation regime at 3 T. The saturation regime may be explained by a low surface contribution and/or weak dipolar magnetic interactions among NPs, as observed by TEM. It is also important to mention that a 4 K FC M­(H) loop, recorded by cooling the sample from 300 K under an applied field of 10 kOe (not shown), shows no horizontal loop shift effect, ruling out a measurable Exchange bias (EB) effect that would occur at the interface between the FM CoO_2_ core and AF CoO shell layer. This apparent absence of the EB effect may be explained by the larger FM/AF ratio (i.e., HEB∝1tFM where *t*_FM_ is the thickness of FM). Summarizing, the low value of H_C_ field (see Figure 6b inset) indicates that the hybrid CoO_2_@CoO presents small dipolar magnetic interactions (supported by TEM image that shows a larger NP dispersion) and behaves as a soft magnetic material.

To have a better understanding of the physical origin of the peak observed at approximately 8 K in the ZFC M­(T) curve recorded at 10 Oe for the hybrid CoO_2_@CoO-NPs, frequency dependent magnetic susceptibility (χ­(*f*,*T*)) experiments were performed, and the results are displayed in Figure 8a for a temperature interval from 2 to 15 K.

**Figure 8:**
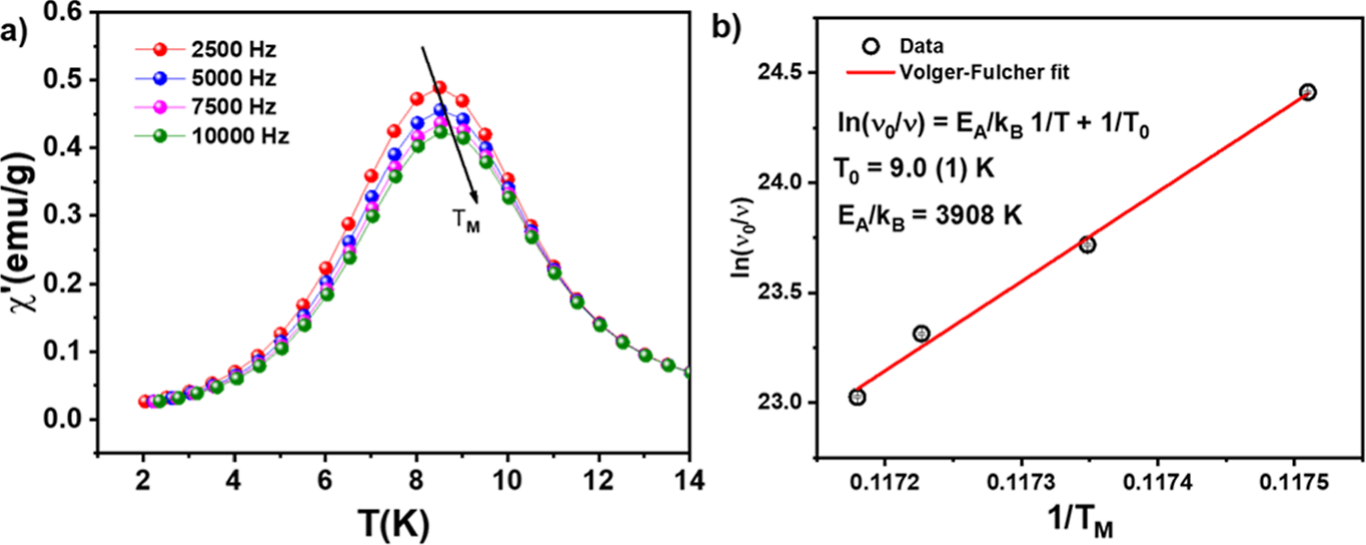
(a) The temperature dependence of the real part of the susceptible alternating current, χ′, measured at different frequencies with an applied alternating field of 10 Oe after ZFC processes. In (b), it is plotted the reduced temperature dependence of the hybrid CoO_2_@CoO-NPs relaxation time *T*_M_ measured at different frequencies. The solid line in (b) is the result of fitting with a linear function given by Volger–Fulcher ln­(ν_0_/ν) = (*k*_ef_ *V*)/*k*_B_(1/*T*_M_).

Figure 8a shows that the peak of χ­(*f*,*T*) slightly shifts to higher temperatures with increasing frequency, a behavior observed in both spin-glass-like (SG) materials and in small magnetic particles.^28,29^ For an ensemble of noninteracting magnetic nanoparticles (as expected for our NPs), the Arrhenius-type model, described by eq 3, can be applied.^30^ For an ensemble of magnetic particles with a narrower magnetic size distribution, *E*_anis_ = *K*_ef_ *V*, where *K*_ef_ is the effective magnetic anisotropy and *V* their average size (≈8 nm), previously determined by TEM, for example.

3 $$\tau =\tau_{0}e^{E_{anis}/k_{B}T}$$

4 $$\tau {=\tau }_{0}e^{K_{ef}V/k_{B}T_{M}}$$

In eq 4, τ_o_ is the characteristic relaxation time, which varies from 10^–9^ to 10^–13^ s,^30^ *K*_ef_ *V* is the magnetic energy barrier over which the spins of NPs flip from one configuration (spin-up) to another (spin-down) and vice versa. Thus, it depends on *K*_ef_ and the particle volume, *V*. The term *k*_B_ *T*_M_ corresponds to the thermal energy that allows the spins of a specific NP to flip between the two orientations (*k*_B_ = 1.38 × 10^–23^ J K^–1^ and *T*_M_ is the peak temperature of χ curve). In Figure 8b, the plot of ln(ννO) versus $\frac{1}{T_{M}}$ shows a linear behavior. From its slope, a *K*_ef_ = 2.4 × 10^2^ kJ m^–3^ was determined. As shown by the fitting equation $\ln\left(\frac{\nu }{\nu_{0}}\right)=\frac{K_{ef}V}{k_{B}}\frac{1}{T_{M}}$ , it does not cross the origin, indicating a weak magnetic interaction among the NPs and/or a contribution from the particle surface that may exhibit a SG-like contribution, in accordance with the weak shift of the susceptibility peak with frequency (Figure 8a).

In light of the above, magnetic properties in a catalyst provide a key practical advantage in heterogeneous reactions. The catalyst can be easily recovered from the reaction medium using an external magnetic field, avoiding complex separation steps and reducing material loss. This facilitates reuse, lowers operational costs, and improves process sustainability. Therefore, combining catalytic activity with magnetic responsiveness makes these materials especially attractive for efficient and recyclable catalytic systems.

### 3.3 Catalytic Activity

Hydrogen generation from NaBH_4_ solutions promoted by metal nanoparticles has been studied in recent years.^31–33^ The search for catalytic systems that can enhance the hydrolysis rate of NaBH_4_ is gaining relevance, since this rate is significantly slow when carried out without a catalyst, especially in an alkaline medium.^17^ In this context, we evaluated the catalytic activity of the synthesized CoO_2_@CoO-NPs. The influence of key reaction parameters in the hydrogen generation was studied. The effect of catalyst weight on hydrogen production over time is displayed in Figure 9a and the HGR values for each catalyst mass are shown in Figure 9b.

**Figure 9:**
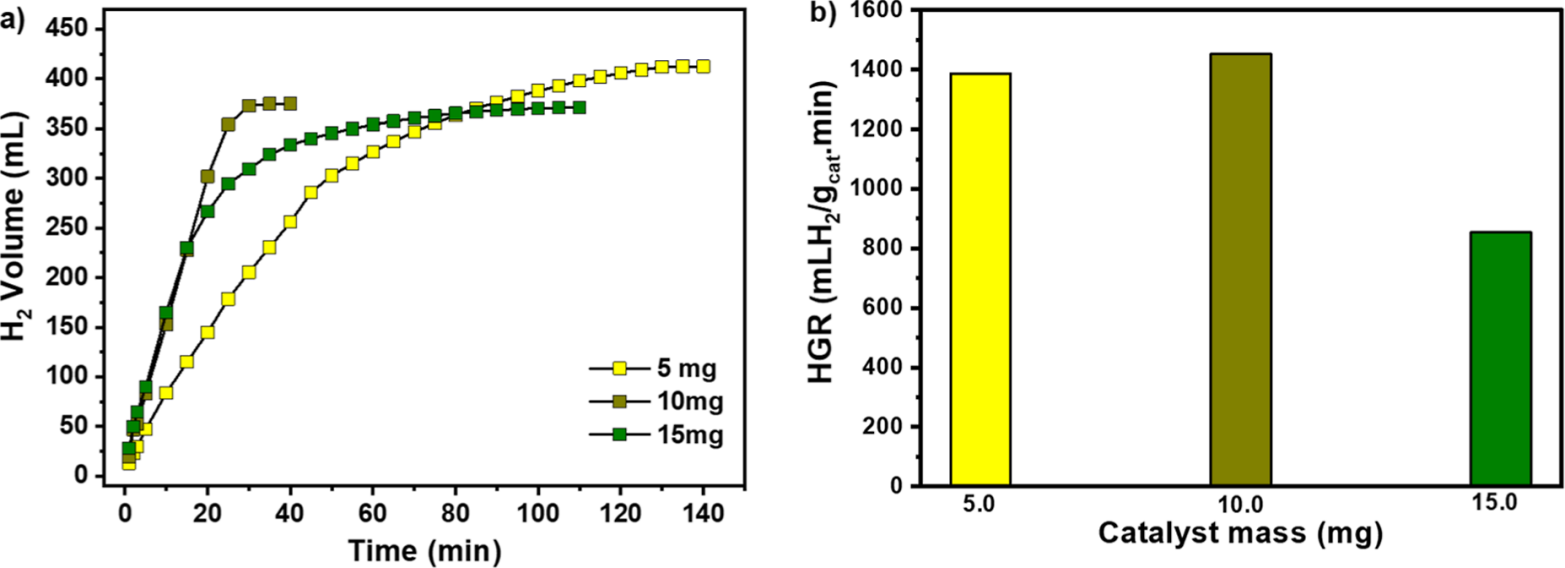
Effect of catalyst amount on hydrogen production. (a) Hydrogen production from NaBH_4_ hydrolysis as a function of time in the presence of different CoO_2_@CoO-NP catalyst masses. (b) Hydrogen generation rate (HGR) obtained with different amounts of the CoO_2_@CoO-NP catalyst. Reaction conditions: 5, 10, and 15 mg of catalyst, respectively, 2 wt % NaBH_4_ concentration, 10 wt % NaOH concentration and 25 °C.

The results show that the time required for the expected volume of hydrogen production decreases with increasing catalyst amount from 5 to 10 mg. The enhanced catalytic performance can be associated with the nature of the surface cobalt species. In particular, surface-exposed Co^2+^ centers in the CoO shell are proposed to act as catalytic sites by facilitating BH_4_^–^ adsorption and promoting B–H bond activation. Furthermore, the nanoscale particle size (∼8 nm) results in a high proportion of low-coordination surface atoms and defect sites, which enhance adsorption and charge-transfer processes. The coexistence of CoO_2_ and CoO phases may additionally induce interfacial electronic modulation, favoring the hydrolysis reaction.^34^ However, this time increases when the catalyst mass is increased to 15 mg. Furthermore, it can be seen that with an increase in the catalyst amount from 5 to 10 mg, the HGR value slightly increases and, when the catalyst amount is increased to 15 mg, the HGR value decreased, indicating that part of the catalyst surface remained uninhabited by the reactant adsorbed species.^35^ With 10 mg the active sites of CoO_2_@CoO NP catalyst appear to be well utilized and, therefore, the following tests were conducted with this mass. With the aim of inhibiting the self-hydrolysis of NaBH_4_ solution, sodium hydroxide is commonly employed as a stabilizing agent.^36^ The influence of the NaOH concentration on hydrogen generation was investigated (Figure 10).

**Figure 10:**
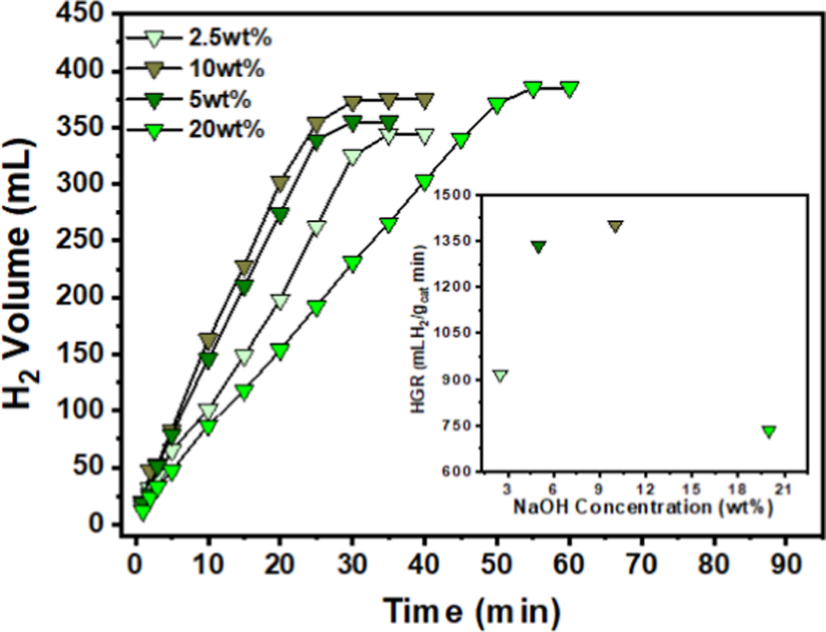
Hydrogen production from NaBH_4_ hydrolysis as a function of time in the presence of different NaOH concentrations. Inset: Hydrogen generation rate (HGR) as a function of NaOH concentration. Reaction conditions: 10 mg of catalyst, 2 wt % NaBH_4_ concentration, 2.5, 5, 10, and 20 wt % NaOH concentration, respectively, and 25 °C.

The results show that hydrogen generation rate (HGR) is greatly favored by increasing the NaOH concentration from 2.5 to 5 wt %, and only slightly increases when the concentration rises to 10 wt %. However, with the NaOH concentration increased to 20 wt %, the hydrogen generation rate decreased significantly, and the time for the complete hydrogen production was extended. There is no consensus regarding the effect of NaOH concentration on hydrogen release during cobalt-catalyzed NaBH_4_ hydrolysis. Conflicting results have been reported to date.^37,38^ Indeed, the beneficial or detrimental effects of the base depend on both its concentration in the reaction medium and the type of catalyst employed.^37,38^ In our case, a NaOH concentration up to 10 wt % proved beneficial for HGR. Possibly, an appropriate OH^–^ concentration favors the dispersion of the hybrid CoO_2_@CoO-NPs in the reaction medium, improving their contact with the NaBH_4_ solution, and thus, enhancing the HGR.^39,40^ Nevertheless, when the base concentration exceeds this value, the performance starts to decrease. When the NaOH concentration exceeds 10 wt %, the decrease in hydrogen generation rate can be attributed to combined bulk-solution and surface effects. In highly alkaline media, the activity of water decreases due to strong hydration interactions between OH^–^ ions and water molecules, reducing the availability of free water required for the hydrolysis reaction.^41^ In parallel, the increased concentration of OH^–^ in solution enhances competitive surface interactions at exposed cobalt sites. Under these conditions, OH^–^ species may coordinate to surface Co^2+^ centers, limiting the interaction of BH_4_^–^ with these catalytic sites and consequently lowering the hydrogen generation rate.^42^ Therefore, both reduced water activity in the bulk solution and competitive surface coordination phenomena contribute to the observed performance decline at elevated NaOH concentrations.^43–46^

To sum up, for some NaBH_4_ hydrolysis process in the presence of Co-based catalysts, there is an optimum NaOH concentration range to improve the hydrogen release. Hydrogen production efficiency at four different NaBH_4_ concentrations was evaluated. The hydrogen volume generated was plotted as a function of time and the graph is depicted in Figure 11a.

**Figure 11:**
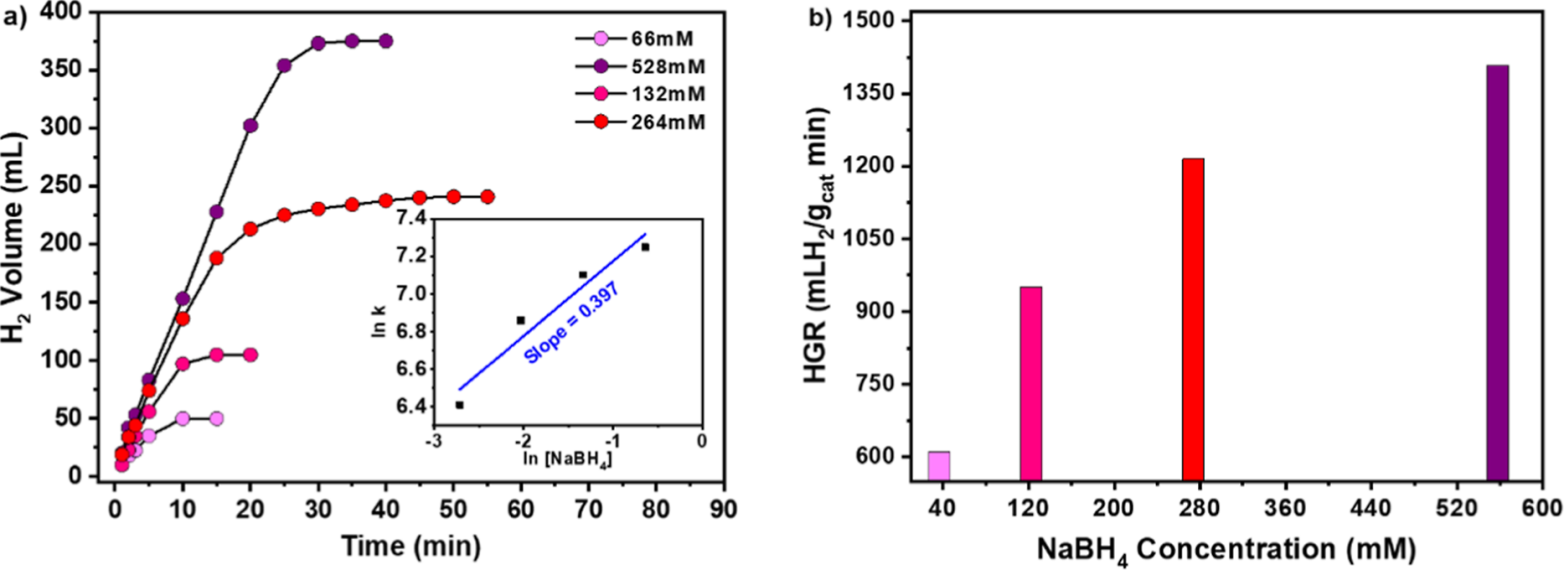
(a) Hydrogen production from NaBH_4_ hydrolysis as a function of time in the presence of different NaBH_4_ concentrations. Inset: plot of ln (hydrogen production rate) vs ln (concentration of NaBH_4_) (b) Hydrogen generation rate (HGR) as a function of NaBH_4_ concentration. Reaction conditions: 10 mg of catalyst, 10 wt % NaOH concentration, 66, 132, 264, and 528 mM NaBH_4_ concentrations, respectively, and 25 °C.

There is an increase in hydrogen production volume as the substrate concentration increases, in accordance with the stoichiometry of the NaBH_4_ hydrolysis reaction. Figure 11b reveals a significant and gradual increase in the HGR as the NaBH_4_ concentration rises up to 264 mM, with the most pronounced effect observed between 66 and 132 mM. However, this trend begins to decelerate when the concentration is further increased to 528 mM. If, on the one hand, from the perspective of chemical equilibrium, an increase in the concentration of NaBH_4_ favors the hydrogen generation, on the other hand, it can lead to the formation of NaBO_2_. Due to the low solubility of the byproduct under alkaline conditions, the viscosity of the solution may increase, causing mass-transfer limitations. Moreover, NaBO_2_ may accumulate on the solid surface, blocking the active catalytic sites.^47,48^ In Figure 11a inset, it is displayed the calculated slope of ln (HGR) (or ln k) versus ln (NaBH_4_) concentration plot to be 0.397, a value that indicates that the reaction order is close to 0.4 according to NaBH_4_ substrate concentration.^49^ Figure 12a shows the effect of reaction temperature on the hydrogen production from NaBH_4_ hydrolysis catalyzed by hybrid CoO_2_@CoO-NPs, keeping the NaOH and NaBH_4_ concentrations kept constant. The rate of the hydrogen production reaction increased drastically with the increase in temperature, reaching the maximum HGR of 7777.1 mLH_2_ min^–1^ g_cat_^–1^ at 55 °C. The activation energy (*E*_a_) of the reaction was determined through the Arrhenius eq (eq 2). The Arrhenius plot, which is ln­(*k*) versus 1/*T*, is depicted in Figure 12b. The *E*_a_ value calculated was 51.73 kJ mol^–1^.

**Figure 12:**
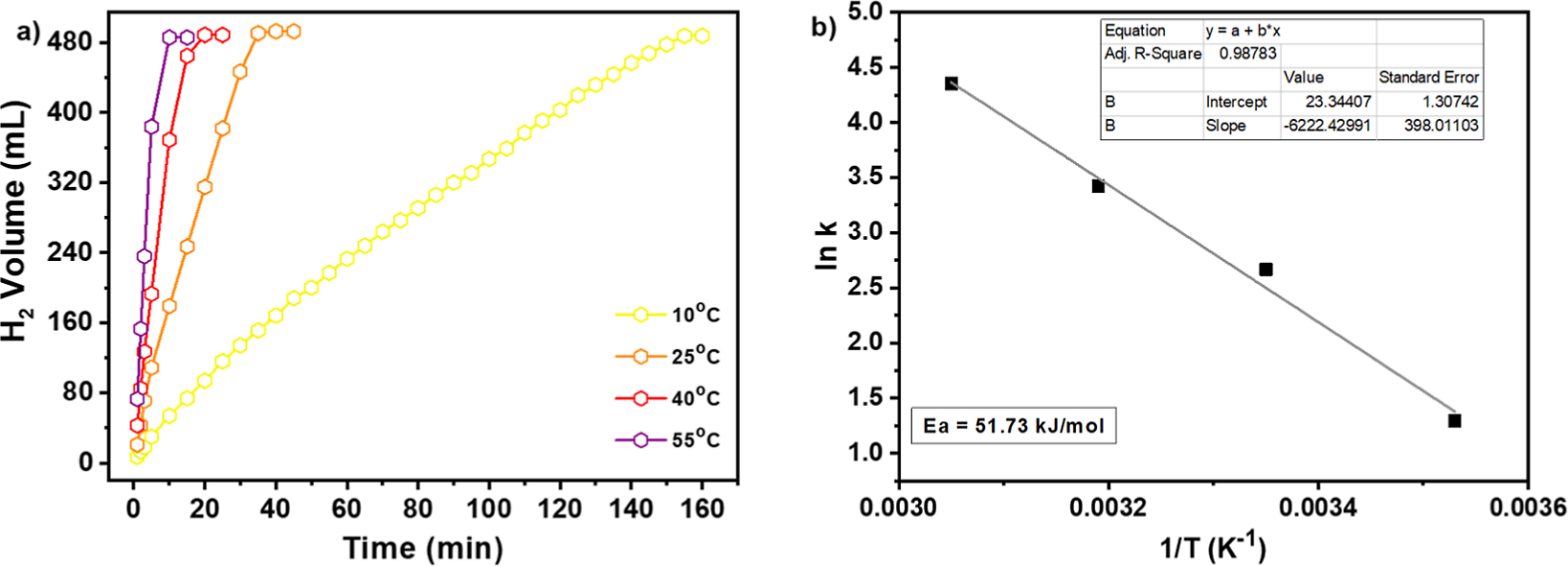
Effect of reaction temperature on hydrogen production. (a) Hydrogen production from NaBH_4_ hydrolysis as a function of time at different temperatures. (b) Arrhenius plot of ln­(*k*) vs the reciprocal absolute temperature (1/*T*). Reaction conditions: 10 mg of catalyst, 2.5 wt % NaBH_4_ concentration, 10 wt % NaOH concentration and 10, 25, 40, and 55 °C, respectively.

With the intention of positioning the hybrid CoO_2_@CoO catalyst performance, a brief compilation of the available catalytic performances was made and the corresponding results are tabulated in Table 1. It is observed that activation energy of the hybrid CoO_2_@CoO catalyst for the hydrolysis of NaBH_4_ is comparable to or lower than previously reported values, and the HGR was also comparable to-or in some cases higher than-that of various previously reported cobalt-based catalysts. Herein, the remarkable HGR and *E*_a_ values can be ascribed to the peculiar morphology of well-dispersed and crystalline nanoparticles obtained, which generate highly accessible active sites.

**Table 1:** Comparison of the Catalytic Performances between Synthesized Hybrid CoO_2_@CoO-NPs and Different Cobalt-Based Catalysts for NaBH_4_ Hydrolysis Previously Reported

catalyst	HGR^a^ (mLH_2_ min^–1^ g_cat_^–1^)	*E*_a_ (kJ mol^–1^)	reference
Co_0.9_Cu_0.1_MoO_4_	1005.7	30.76	^50^
Co–Mo–B/CC	1280.8	51.0	^51^
Co–W–P/γ-Al_2_O_3_	4000	49.58	^52^
CoB–Ni_4_B_3_	500	32.7	^53^
CoS nanoparticles	328	58.8	^54^
Co–Zn–B/graphene	2180	35.92	^55^
CMF-CoO-CoB	6974	35.05	^56^
20 wt %Co/ZX_diatomite	1045	53.45	^57^
Co/TiO_2_(P25)	660	45.2	^58^
Co–Ce–B/Chi-C	4760	33.1	^59^
hybrid CoO_2_@CoO-NPs	1452.5	51.73	This work

a Reaction temperature range = 25–30 °C.

The reusability of the hybrid CoO_2_@CoO catalyst was investigated over three consecutive cycles (Figure 13). As described in the experimental section, the magnetic properties exhibited by the synthesized CoO_2_@CoO-NPs facilitated the separation process for their reuse. The catalytic activity decreased markedly after the use in the first cycle, with HGR changing from 1452.5 mLH_2_ min^–1^ g_cat_^–1^ to 483.2 mLH_2_ min^–1^ g_cat_^–1^, and then, to 260.9 mLH_2_ min^–1^ g_cat_^–1^ in the second and third cycles, respectively. This behavior is likely due to the precipitation of NaBO_2_, which blocks the catalyst’s active sites.^60^ In addition, the transformation of the hybrid CoO_2_@CoO species in the catalyst due to the reaction conditions, particularly the high pH, weakens the catalytic sites, potentially accounting for the observed loss of catalytic activity.^61^

**Figure 13:**
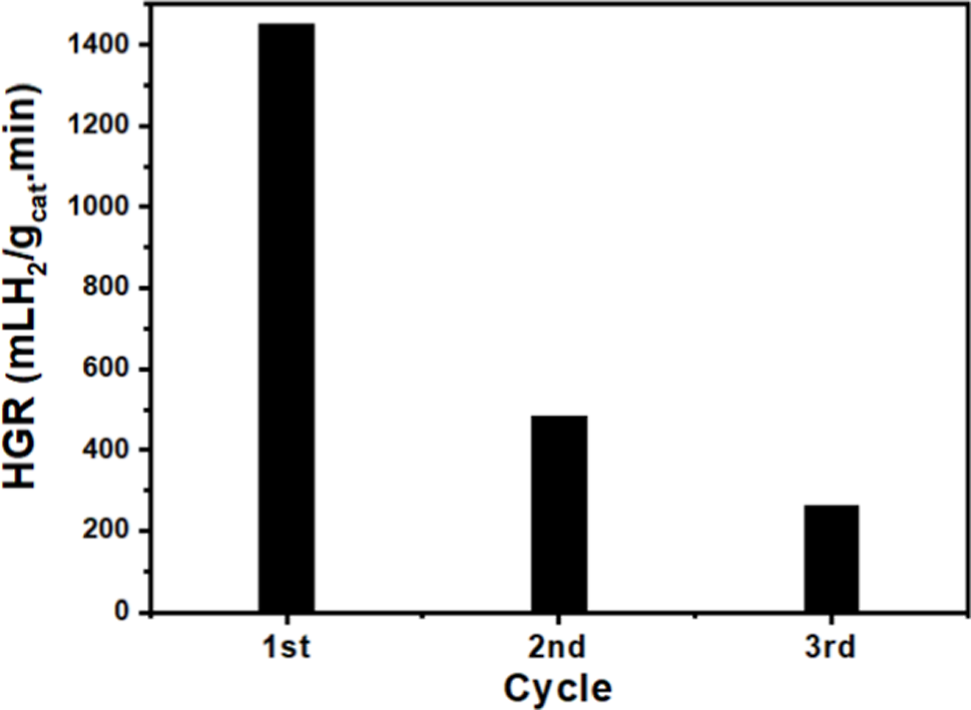
Reusability performance of hybrid CoO_2_@CoO-NPs catalyst expressed by hydrogen production rate (HGR) as a function of consecutive cycles. Reaction conditions: 10 mg of catalyst, 2 wt % NaBH_4_ concentration, 10 wt % NaOH concentration and 25 °C.

## 4 Conclusions

We obtained stable and well-defined hybrid cobalt oxide nanoparticles [CoO_2_@CoO-NPs] using d-Glucose as a stabilizing ligand. The use of this biomolecule, which is very abundant in nature, enables a sustainable and economically attractive synthesis. These hybrid CoO_2_@CoO-NPs are ferromagnetically ordered below 8 K, with a magnetic moment of 9.11 × 10^3^ μ_B_ and an effective anisotropy constant of 2.4 × 10^2^ kJ m^–3^. We evaluated the ability of these hybrid CoO_2_@CoO-NPs to act as catalysts for H_2_ generation via NaBH_4_ hydrolysis. They exhibited excellent catalytic activity due to highly dispersed active sites, as evidenced by an activation energy of 51.73 kJ mol^–1^ and an HGR of 1452.5 mLH_2_ min^–1^ g_cat_^–1^ in the temperature range of 25–30 °C. The catalytic evaluation allowed the identification of well-defined optimal operating conditions, under which maximum efficiency was achieved. Deviations from these conditions led to measurable kinetic limitations: excess NaOH concentrations promoted inhibitory effects associated with OH^–^ competitive adsorption and decreased water activity, whereas increasing catalyst loading beyond the optimal amount resulted in substrate-limited behavior. These findings demonstrate that catalytic performance is governed by a delicate balance between surface availability and reaction medium composition. Overall, this study establishes a simple and cost-effective methodology for obtaining promising catalysts that may offer advantages for hydrogen generation.
